# *In vivo* CAR T-cell generation: delivery platforms, clinical progress, and translational barriers

**DOI:** 10.3389/fimmu.2026.1927594

**Published:** 2026-09-04

**Authors:** Tianqun Huo, Hongping Yao, Ziyan Kong

**Affiliations:** 1 Department of Orthopaedics, Changzhou Hospital Affiliated to Nanjing University of Chinese Medicine, Changzhou, Jiangsu, China; 2 State Key Laboratory of Digital Medical Engineering, School of Biological Science and Medical Engineering, Southeast University, Nanjing, China

**Keywords:** clinical translation, gene delivery, *in vivo* CAR T-cell therapy, lentiviral vectors (LVs), lipid nanoparticles

## Abstract

Chimeric antigen receptor (CAR) T-cell therapy has transformed the treatment of several hematological malignancies, but its broader application remains constrained by the complexity, cost, and time required for conventional ex vivo manufacturing. *In vivo* CAR T-cell therapy has emerged as a promising next-generation strategy that aims to generate CAR T cells directly within the patient through targeted delivery of CAR-encoding genetic information to endogenous T cells. This approach has the potential to simplify treatment workflows, shorten manufacturing timelines, reduce production costs, and improve the accessibility of CAR-based immunotherapy. In this review, we summarize the conceptual evolution from ex vivo to *in vivo* CAR T-cell therapy and discuss major delivery platforms for *in vivo* CAR T-cell generation, including engineered lentiviral vectors (LVs), adeno-associated viral vectors, lipid nanoparticles, polymeric nanoparticles, extracellular vesicles, and fusogenic nanovesicles. We further examine key translational challenges and corresponding optimization strategies, including approaches to improve T-cell targeting specificity and delivery controllability, reduce vector immunogenicity, enhance CAR expression persistence, mitigate safety concerns associated with ectopic transduction or genomic integration, and potentially overcome the physical, antigenic, and immunosuppressive barriers encountered in solid tumors. Finally, we summarize early clinical trial progress and discuss future directions for improving the safety, efficacy, and translational potential of *in vivo* CAR T-cell therapy. Overall, *in vivo* CAR T-cell therapy represents an important extension of adoptive cell therapy and may reshape the development and clinical implementation of cell-based immunotherapies.

## 1 Introduction

Since the introduction of chimeric antigen receptors (CARs) to redirect T-cell antigen recognition, CAR T-cell therapy has emerged as a major breakthrough in cancer immunotherapy (1, 2). Unlike T cell receptor (TCR)-engineered T cells, which depend on major histocompatibility complex (MHC)-mediated antigen presentation, CARs enable T cells to recognize target antigens in an MHC-independent manner, thereby broadening their therapeutic applicability (3). This strategy has been particularly successful in hematologic malignancies such as leukemia and lymphoma, ultimately leading to the regulatory approval of multiple CAR T-cell products (4, 5). Currently, clinical CAR T-cell therapy predominantly relies on an ex vivo manufacturing paradigm: autologous T cells are isolated from patients, activated, expanded, and genetically engineered to express CARs before being reinfused into patients to mediate antigen-specific elimination of target cells (6, 7). This paradigm has established CAR T cells as a “living drug” modality and provided an important foundation for the development of personalized cell-based immunotherapy (8–10).

However, the patient-specific ex vivo manufacturing process poses substantial manufacturing and translational challenges. Complex production procedures, lengthy turnaround times, high costs, and stringent quality control requirements collectively limit the accessibility of CAR T-cell therapy (11, 12). Moreover, prolonged ex vivo manipulation and expansion can compromise T-cell differentiation status, functional fitness, and final product consistency, thereby contributing to variability in therapeutic efficacy and treatment outcomes (13, 14). To overcome these limitations, the CAR T-cell field is undergoing a gradual technological shift from the “cell-as-drug” paradigm toward a “patient-as-bioreactor” concept (9, 15). This has given rise to *in vivo* CAR T-cell therapy, the core concept of which is to directly reprogram endogenous T cells within the body through targeted delivery of genetic information, thereby endowing them with predefined antigen recognition and effector functions (16, 17). Compared with conventional ex vivo approaches, *in vivo* CAR T-cell therapy has the potential to streamline treatment procedures, shorten the time to treatment, lower manufacturing costs, and reduce manufacturing-related challenges associated with ex vivo cell production, including the need for leukapheresis, *in vitro* T-cell activation and expansion, prolonged manufacturing timelines, and variability in product quality (18, 19). With continued advances in delivery platforms and immunoengineering technologies, *in vivo* CAR T-cell therapy is increasingly viewed as a promising strategy for expanding CAR-based immunotherapy to a broader spectrum of diseases, including malignancies, autoimmune disorders, and other immune-related conditions (20–23).

This review systematically outlines the conceptual foundations, delivery strategies, key challenges, and recent advances in the clinical translation of *in vivo* CAR T-cell therapy. We first briefly review the evolution of the CAR T-cell therapeutic paradigm and the emergence of the *in vivo* engineering concept, and then compare the technical principles, key attributes, and translational prospects of major delivery platforms, including viral and non-viral vectors. On this basis, we further summarize the key issues affecting *in vivo* CAR T-cell generation, functional maintenance, and safe clinical translation, and discuss corresponding optimization strategies. Finally, we summarize the current status of early-phase clinical studies in this field and discuss the insights they provide, aiming to offer a clear analytical framework for the transition of *in vivo* CAR T-cell therapy from technological exploration to clinical application.

## 2 Development of CAR T-cell therapy: from ex vivo to *in vivo* engineering

**Figure 1:**
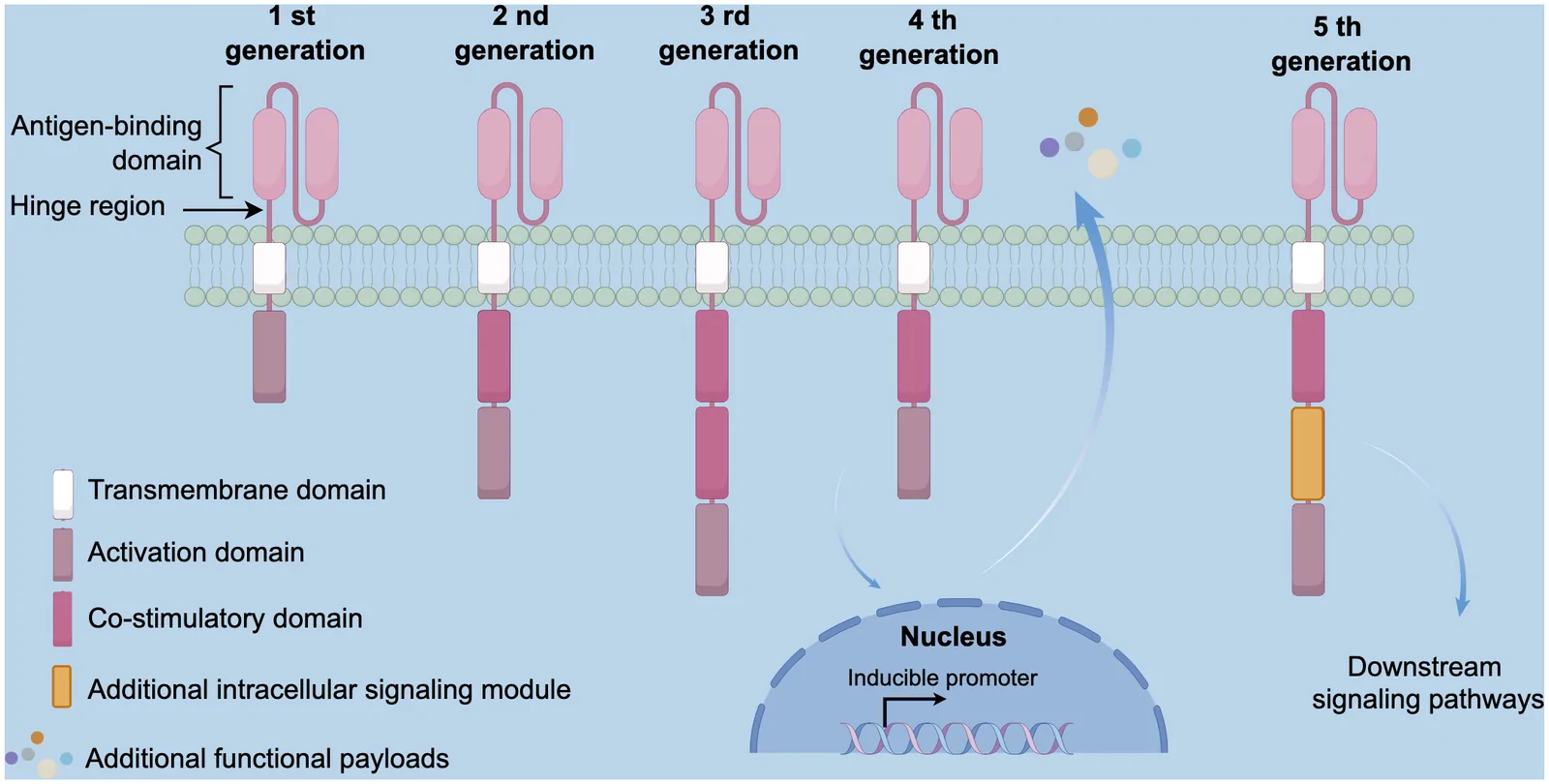
Representative structures and functional evolution of first- through fifth-generation chimeric antigen receptors (CARs). A typical CAR consists of an extracellular antigen-binding domain, a hinge region, a transmembrane domain, and intracellular signaling domains. First-generation CARs contain only an activation domain, typically derived from CD3ζ or FcRγ. Second-generation CARs incorporate a single co-stimulatory domain, such as CD28 or 4-1BB, whereas third-generation CARs contain multiple co-stimulatory domains to enhance T-cell activation and persistence. Fourth-generation CARs, also known as T cells redirected for universal cytokine killing (TRUCKs), incorporate inducible expression systems that enable the release of additional functional payloads, including immunomodulatory cytokines (e.g., IL-12, IL-15, and IL-18), chemokines, enzymes, antibodies, or scFvs upon antigen stimulation. Fifth-generation CARs incorporate additional intracellular signaling modules, such as cytokine receptor-derived signaling elements (e.g., IL-2Rβ-containing domains), which provide additional signaling inputs beyond conventional CAR activation signals, including cytokine receptor-associated pathways such as JAK–STAT signaling.

The development of CAR T-cell therapy has evolved from the initial conception of chimeric receptors, through continuous structural refinement, to the clinical maturation of the ex vivo therapeutic paradigm (24). In the late 1980s and early 1990s, Eshhar et al. first designed chimeric receptors that endowed T cells with antibody-like antigen recognition, laying the groundwork for subsequent advances (25). A typical CAR comprises four domains—an antigen-binding domain, a hinge region, a transmembrane domain, and an intracellular signaling domain—each with distinct functions (26). Based on CAR structural and functional evolution, CARs are commonly categorized as first- through fifth-generation designs. First-generation CARs combine an antigen-recognition domain with a single intracellular activation signal, often derived from the Fc receptor γ chain (FcRγ) or CD3ζ (27). CD3ζ contains multiple immunoreceptor tyrosine-based activation motifs (ITAMs) and can mediate potent T-cell activation (28). However, the lack of co-stimulatory signaling limits the *in vivo* expansion, persistence, and antitumour activity of first-generation CAR T cells. Second-generation CARs add a co-stimulatory domain such as CD28 or 4-1BB to CD3ζ, markedly improving proliferation, persistence, and effector function. This architecture forms the principal design basis of currently approved CAR T-cell products (29–31). Subsequent CAR designs have further evolved toward functional enhancement and more refined control. Third-generation CARs incorporate two co-stimulatory domains to synergistically enhance T-cell activity and persistence, although consistent clinical superiority over second-generation designs has not been established (32, 33). Fourth-generation CARs, including T cells redirected for universal cytokine killing (TRUCKs), can be engineered to express immunomodulatory cytokines, such as IL-12, IL-15, and IL-18, thereby enhancing CAR T-cell persistence, immune activation, and antitumor responses within the tumor microenvironment (34–36). In TRUCK designs, inducible expression systems, such as nuclear factor of activated T cells (NFAT)-responsive promoters, enable antigen-triggered local cytokine release (34). Fifth-generation CARs further couple cytokine-receptor-associated signaling modules—for example, a truncated IL-2Rβ cytoplasmic domain and STAT3-recruiting motifs—allowing antigen recognition to synergize with JAK–STAT signaling and thereby supporting CAR T-cell expansion, functional maintenance, and *in vivo* persistence (37, 38). The structural evolution and representative features of first- through fifth-generation CARs are summarized in **Figure 1**.

A milestone was reached in 2011, when CAR T-cell therapy achieved landmark clinical advances in hematologic malignancies, sparking widespread interest in product development (39). Several CAR T-cell products are now approved for B-cell malignancies and multiple myeloma, substantially reshaping the therapeutic landscape for some patients with relapsed or refractory disease. Beyond oncology, CAR T-cell therapy has also shown notable potential in various refractory non-malignant diseases and other conditions with substantial unmet therapeutic needs (40, 41). All currently approved CAR T-cell products are autologous and are generated through ex vivo engineering of a patient’s own T cells. The typical manufacturing process involves: (a) collecting peripheral blood mononuclear cells or T cells by leukapheresis; (b) activating T cells ex vivo and introducing the CAR transgene using viral or non-viral delivery systems; (c) expanding the transduced T cells; (d) concentrating and collecting the CAR T cells; and (e) reinfusing the final product into the patient (42, 43). This complex workflow—together with high costs, the clinical risks associated with lymphodepleting preconditioning, and T-cell exhaustion—severely constrains the broader implementation and equitable access of CAR T-cell immunotherapy (44, 45).

**Figure 2:**
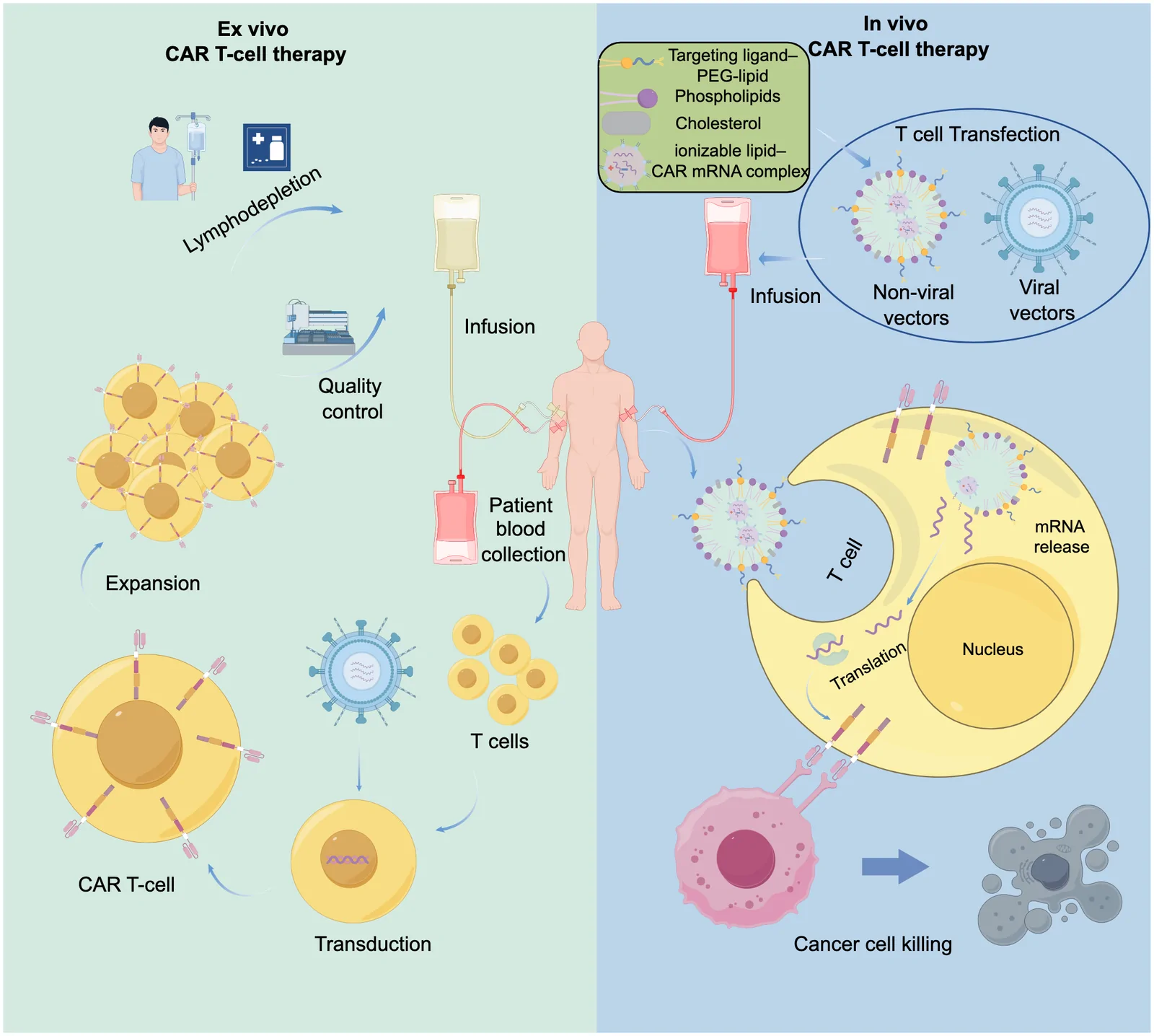
Schematic comparison of conventional ex vivo CAR T-cell therapy and *in vivo* CAR T-cell therapy. The ex vivo approach relies on leukapheresis, genetic engineering, expansion, quality control, and reinfusion of autologous CAR T cells, whereas the *in vivo* strategy directly generates CAR T cells within the patient primarily through targeted delivery of CAR-encoding genetic information or CAR molecules. The figure highlights differences in workflow, manufacturing complexity, treatment timeline, and translational challenges.

Against this backdrop, and propelled by the convergence of advances in gene delivery, RNA therapeutics, and immune-cell engineering, *in vivo* CAR T-cell generation has emerged as a research frontier (46). The key differences between ex vivo and *in vivo* CAR T-cell therapy, including workflow, manufacturing features, and translational characteristics, are summarized in **Figure 2**. Mechanistically, both *in vivo* and conventional ex vivo CAR T-cell therapies rely on CAR-mediated T-cell redirection and cytotoxicity. However, *in vivo* manufacturing strategies may help address several limitations of ex vivo CAR T-cell therapy. These strategies also offer unique advantages in standardized manufacturing, ease of transport, and rapid clinical deployability (47). Notably, several *in vivo* CAR T-cell therapies have entered clinical trials, with ongoing advances in T-cell-targeted delivery, *in vivo* transduction efficiency, and expression control (18). Their clinical exploration has also expanded from malignancies to autoimmune diseases and other refractory conditions, pointing to broader therapeutic potential (22, 23).

## 3 Approaches for *in vivo* CAR T-cell generation

The realization of *in vivo* CAR T-cell therapy depends on the efficient and selective delivery of CAR transgenes or CAR-encoding nucleic acids to target T cells within the body, thereby inducing CAR T cells with defined antigen-recognition and effector functions *in situ* (48, 49). To achieve this goal, researchers have developed various delivery and engineering strategies, which can be broadly divided into two main categories: viral vectors and non-viral delivery systems. Viral vectors, represented by retroviruses, lentiviruses, and adeno-associated viruses (AAVs), generally exhibit high transduction efficiency (50, 51). Non-viral systems encompass lipid nanoparticles (LNPs), polymeric nanoparticles, extracellular vesicles (EVs), and fusogenic nanovesicles, and have attracted considerable attention owing to their design flexibility, capacity for non-integrating nucleic acid delivery, and manufacturing scalability (52, 53). Below, we outline the design principles, representative advances, and key application features of these platforms in the context of *in vivo* CAR T-cell generation.

### 3.1 Viral vectors

Current viral delivery systems for the *in situ* generation of CAR T cells mainly include lentiviral vectors (LVs) and AAVs. LVs are the most widely used gene delivery tools and have been extensively employed for the ex vivo manufacturing of clinical-grade CAR T cells (54). Their advantages include stable integration of relatively large exogenous DNA sequences into the host genome and high transduction efficiency in both dividing and non-dividing cells (55, 56). Moreover, pseudotyping with envelope proteins such as VSV-G confers broad cell tropism (57). However, conventional LVs lack cell-type specificity, making selective *in vivo* delivery to T cells challenging. To improve targeting, researchers often use envelope pseudotyping strategies to replace native envelope proteins with engineered ligands — such as single-chain variable fragments (scFvs) targeting CD3, CD4 or CD8 — thereby enabling T-cell-specific recognition (57–60). An ideal vector for *in vivo* CAR T-cell generation should possess: (1) low off-target effects; (2) high T-cell specificity; and (3) efficient gene delivery (61). Michels and colleagues developed VivoVec, an *in vivo* T-cell engineering platform based on LVs, and demonstrated its feasibility for generating CAR T cells directly *in vivo* in preclinical models (62). This platform enhances T-cell binding and transduction by displaying T-cell-targeting molecules on the viral surface. Subsequent optimization of the VivoVec platform further incorporated multi-domain fusion (MDF) proteins, in which an anti-CD3 single-chain variable fragment (scFv) is combined with co-stimulatory molecules such as CD80 and CD58 on the viral particle surface. This design enables the vector to engage T cells while simultaneously providing initial activation and co-stimulatory signals, thereby enhancing CAR T-cell generation and supporting prolonged CAR T-cell persistence *in vivo* (63). In addition, the VivoVec platform incorporates cytokine-like stimulation through a rapamycin-activated cytokine receptor (RACR) system, which provides IL-2/IL-15 signaling to enhance CAR T-cell expansion and functional persistence, reducing dependence on conventional lymphodepleting preconditioning and potentially simplifying CAR T-cell manufacturing and treatment workflows (62, 64). In a complementary approach, Andorko et al. engineered VSV-G through site-specific mutations to abolish binding to its native receptor LDL-R while preserving membrane-fusion activity. When combined with targeting ligands against CD3, CD4, or CD7, this modified envelope system enabled selective transduction of specific immune-cell populations. This strategy enhances targeting while reducing the risk of non-specific transduction, offering another feasible route for *in vivo* CAR immune-cell engineering (65).

On the other hand, AAVs have also been extensively explored for *in situ* generation of *in vivo* CAR T cells owing to their favorable safety profile and stable expression capacity. AAVs are non-enveloped, single-stranded DNA viruses with a genome of approximately 4.7 kb. The transgenes delivered by AAV typically persist as episomes in host cells, enabling relatively durable expression while reducing risks associated with genomic integration (66, 67). Compared with adenoviruses, AAVs have lower immunogenicity, and recombinant AAVs lack viral replication and pathogenicity-related genes, contributing to their favorable safety profile in clinical applications (68, 69). In preclinical studies of *in vivo* CAR T-cell generation, Nawaz et al. achieved reprogramming of immune cells in mice using modified AAV vectors, leading to CAR expression and effective tumour growth suppression (70). Furthermore, capsid engineering — using chimeric serotypes such as AAV-DJ — can further optimize cell tropism and transduction efficiency, thereby improving T-cell transduction capacity (70).

Despite the promise of viral vectors for *in vivo* CAR T-cell engineering, their clinical translation faces multiple challenges. The integrating nature of LVs carries a risk of insertional mutagenesis, and non-specific delivery may lead to ectopic CAR expression in non-target cells (71). In addition, host immune responses against viral vectors may limit delivery efficiency and durability (72). Overall, improving T-cell-specific delivery efficiency while minimizing immunogenicity and genome-related risks remains a key challenge in viral vector-mediated *in vivo* CAR T-cell generation.

### 3.2 Non-viral vectors

Compared with viral vectors, non-viral delivery systems offer greater design flexibility, lower immunogenicity and superior manufacturing scalability. Based on their material origin and delivery strategy, non-viral vectors currently used for *in vivo* CAR engineering can be broadly classified into synthetic nanoparticles and bioinspired nanoparticles.

#### 3.2.1 Synthetic nanoparticles

##### 3.2.1.1 Lipid nanoparticles

Lipid nanoparticles (LNPs) are among the most widely used non-viral delivery platforms for *in vivo* CAR T-cell engineering, enabling efficient nucleic acid delivery to target cells while preventing rapid degradation or clearance *in vivo* (73). Compared with viral vectors, LNPs offer greater engineering flexibility — including surface modification for T-cell targeting — along with improved safety, lower production costs and superior scalability (74, 75). Moreover, nucleic acid payloads, particularly mRNA, delivered by LNPs are typically expressed transiently in the cytoplasm, thereby avoiding the risk of insertional mutagenesis associated with genomic integration and potentially reducing the risks of long-term CAR-related toxicity and cytokine release syndrome (CRS) (76, 77). LNPs are therefore considered one of the most promising delivery strategies for clinical translation of *in vivo* CAR T-cell therapy. LNPs can efficiently encapsulate various nucleic acid molecules — including mRNA, DNA and CRISPR gene-editing components — forming stable nanostructures that enhance *in vivo* stability and delivery efficiency (78, 79). A typical LNP consists of four core components: ionizable lipids, phospholipids, cholesterol and polyethylene glycol (PEG)-modified lipids (80). In addition to ionizable lipids, phospholipids provide structural support to the nanoparticles, cholesterol contributes to membrane stability and lipid organization, while PEG-modified lipids help reduce particle aggregation and improve colloidal stability (80). The ionizable lipids become positively charged under acidic conditions, facilitating nucleic acid encapsulation and endosomal escape, while remaining near-neutral under physiological conditions, thereby reducing systemic toxicity. During delivery, LNPs typically enter target cells via receptor-mediated endocytosis and achieve endosomal escape through a pH-responsive mechanism, ultimately releasing nucleic acids into the cytoplasm, where mRNA can be rapidly translated into protein (81, 82). The relatively straightforward *in vitro* synthesis of mRNA also circumvents the complex manufacturing processes required for viral vectors, further reducing production costs.

Multiple studies have validated the feasibility of LNP-mediated *in vivo* CAR T-cell generation. For example, Rurik et al. used CD5-targeted LNPs to deliver mRNA encoding a fibroblast activation protein (FAP)-specific CAR, resulting in a significant reduction in cardiac fibrosis and improved cardiac function in a mouse model of heart failure (83). Similarly, Li et al. generated *in vivo* CAR T cells by co-delivering mRNAs encoding a CAR and IL-7, leading to effective melanoma cell clearance and suggesting that cytokine co-expression strategies may further enhance therapeutic efficacy (84). Beyond conventional mRNA, researchers are also exploring alternative nucleic acid payloads to improve the stability and durability of CAR expression. Compared with linear mRNA, circular RNA (circRNA) offers substantially greater stability owing to its closed-loop structure, enabling more sustained protein expression (85). Wang et al. reported that LNP-delivered circRNA encoding a CAR maintained CAR expression for more than 72 hours *in vivo* and effectively suppressed tumour growth (86). In addition, DNA-based delivery strategies have been explored to achieve more durable CAR expression; for example, co-delivery of minicircle DNA with transposase mRNA enables stable genomic integration of the CAR gene in T cells via transposon-mediated mechanisms (87). The clinical feasibility of LNP delivery platforms has also been validated — Patisiran, as the first FDA-approved LNP-formulated siRNA therapeutic, provides an important reference for the clinical translation of this platform (88).

Current LNP optimization efforts focus primarily on two aspects: targeting modifications and regulation of physicochemical properties. On one hand, coupling anti-CD3 or anti-CD8 single-chain variable fragments (scFvs), nanobodies (e.g., Nb8-H3), or designed ankyrin repeat proteins (DARPins) to the LNP surface enhances specific recognition of T cells and facilitates receptor-mediated endocytosis (52, 88–92). On the other hand, tuning parameters such as particle size (typically optimized to less than 100 nm), PEG lipid dissociation rate and targeting ligand density can further improve *in vivo* delivery efficiency and cellular uptake (93–96). Thus, systematic optimization of LNP composition, structural characteristics and surface modification to achieve efficient and precise T-cell delivery remains an important research direction for *in vivo* CAR T-cell engineering.

##### 3.2.1.2 Polymeric nanoparticles

Compared with liposomes, polymeric nanoparticles offer greater structural diversity and are more readily modified, enabling precise control over particle size and morphology (97). These properties confer controllable drug-loading capacity and superior delivery performance. In gene delivery, polymeric nanoparticles form nucleic acid complexes through electrostatic interactions. Compared with viral vectors, some biodegradable polymer systems based on this binding mechanism exhibit lower immunogenicity, can be structurally optimized to reduce toxicity, protect nucleic acids from nuclease degradation, and promote efficient intracellular delivery and release (75, 98). However, compared with LNPs, polymeric nanoparticles remain less clinically mature for nucleic acid delivery, and their delivery performance can vary substantially with polymer structure and nucleic acid cargo (99, 100). Moreover, the strong dependence of transfection efficiency and toxicity on polymer molecular weight, sequence, end groups, and topology increases formulation complexity, while conventional preparation methods for polymeric nanoparticles may result in batch-to-batch variability and limited scalability (100, 101). Such considerations are particularly relevant to *in vivo* CAR-T applications, which require efficient and reproducible T-cell engineering. Despite these challenges, polymeric nanoparticles have shown promising potential for *in vivo* T-cell engineering.

Smith et al. first attempted to use polymeric nanoparticles to deliver nucleic acids to T cells *in vivo*. They encapsulated plasmid DNA encoding a leukemia-specific CAR in cationic poly (β-amino ester) (PBAE) nanoparticles, thereby achieving *in vivo* engineering of T cells. In subsequent studies, the same group constructed PBAE nanoparticles encapsulating mRNA encoding prostate tumor-specific CAR or leukemia-associated CARs, and systemic administration of these nanoparticles elicited robust antitumor responses in mice (73, 92).

#### 3.2.2 Bioinspired nanoparticles

##### 3.2.2.1 Extracellular vesicles

Extracellular vesicles (EVs), particularly exosomes, have emerged as promising bioinspired nanocarriers for *in vivo* immune cell engineering. Exosomes are naturally secreted nanoscale vesicles, typically 40–160 nm in diameter, that mediate intercellular communication by delivering proteins, lipids, and nucleic acids (102). Owing to their preservation of native cell membrane characteristics, exosomes offer superior biocompatibility, enhanced stability, lower immunogenicity and intrinsic tissue tropism compared with conventional synthetic nanocarriers, positioning them as attractive platforms for *in vivo* delivery of CAR-related genetic material (103). Recent studies have demonstrated the utility of exosomes for direct *in vivo* CAR T-cell generation. For example, researchers engineered an exosome platform displaying anti-CD3/CD28 scFvs on the surface for T-cell targeting, while efficiently encapsulating CAR mRNA using the bacteriophage MS2 system together with the cargo-loading protein LAMP-2B (104). This strategy provides a new approach for the *in vivo* generation of CAR T cells.

Beyond direct CAR gene delivery, exosomes have also been developed as immunotherapeutic platforms that mimic CAR T-cell function. For instance, tumor antigen-stimulated dendritic cell-derived exosomes (tDC-Exo) were engineered to express anti-CD3 and anti-EGFR molecules (105). This platform not only activated endogenous T cells but also promoted T cell–tumor cell engagement, thereby enhancing antitumor immune responses. Despite their promising potential, clinical translation of exosome delivery systems faces several challenges, including limited cargo encapsulation efficiency, heterogeneity of vesicle composition, difficulties in large-scale production, and lack of standardized purification protocols (106, 107). Further optimization of engineering strategies is needed to advance the clinical application of exosomes in *in vivo* CAR T-cell therapy.

##### 3.2.2.2 Fusogenic nanovesicles

Conventional non-viral delivery systems typically rely on intracellular transcription or translation following nucleic acid delivery, but limited endocytosis efficiency and insufficient endosomal escape often constrain delivery performance. To overcome these limitations, fusogenic nanovesicles (FNVs) have gained attention as a novel bioinspired delivery platform, distinguished by their ability to deliver pre-assembled CAR proteins directly through membrane fusion, thereby bypassing the need for nucleic acid delivery (108–110). A recent study developed virus-mimetic fusogenic nanovesicles (FuNVs) by co-expressing CAR proteins together with fusogens derived from reovirus or measles virus in donor cells, further incorporating anti-CD3 scFvs for T-cell targeting (111). This platform directly fuses with the T-cell membrane to deliver preassembled CAR proteins onto the T-cell surface, thereby rapidly generating functional CAR-expressing T cells *in vivo* and demonstrating robust antitumor activity in a CD19-positive B-cell lymphoma model. Building on this approach, T cell-targeted fusogenic nanovesicles were further developed for the treatment of rheumatoid arthritis (110). This strategy similarly generated anti-CD19 CAR T cells *in vivo* through direct membrane fusion, depleting pathogenic B cells and significantly alleviating disease progression in a collagen-induced arthritis model. Collectively, fusogenic nanovesicles offer a distinct non-genetic strategy for *in vivo* CAR engineering through direct CAR protein delivery. However, this platform still faces challenges including limited duration of CAR expression, potential requirement for repeated administration, and difficulties in large-scale manufacturing. Moreover, given the transient nature of CAR protein expression, the utility of this strategy in solid tumors that require sustained immunological surveillance warrants further validation (108).

## 4 Challenges and potential enhancement strategies

**Figure 3:**
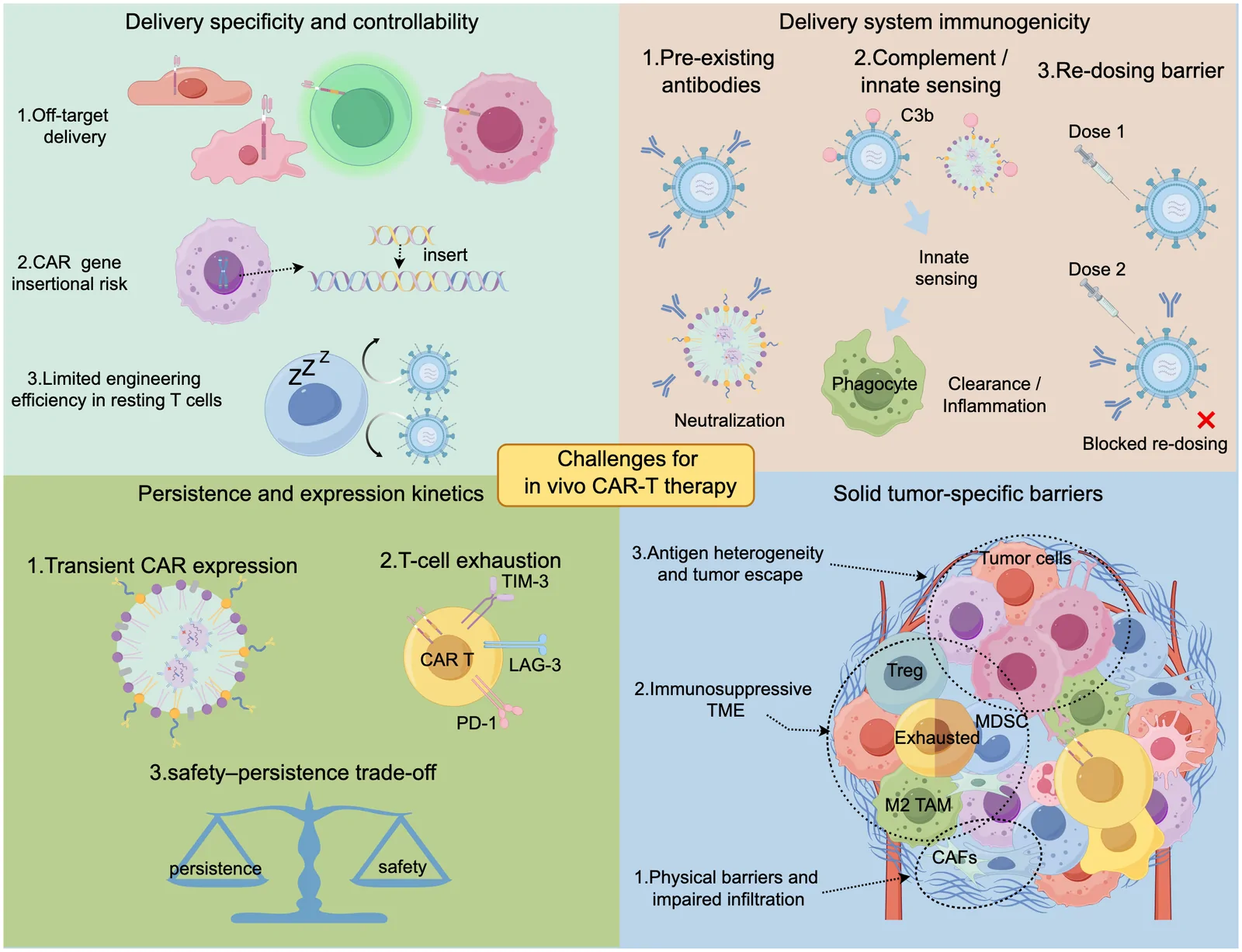
Major challenges limiting the clinical translation of *in vivo* CAR T-cell therapy. Key challenges include limited delivery specificity and engineering controllability, delivery system immunogenicity and repeat dosing barriers, suboptimal persistence and expression kinetics, and solid tumor-specific barriers. Together, off-target delivery, inefficient engineering of resting T cells, immune recognition of viral or non-viral delivery systems, transient or uncontrolled CAR expression, T-cell exhaustion, impaired tumor infiltration, immunosuppressive tumor microenvironmental factors and antigen heterogeneity collectively restrict the safety, efficacy, and durability of *in vivo*-generated CAR T cells.

*In vivo* CAR T-cell therapy — which eliminates the need for ex vivo cell isolation, genetic modification and expansion — offers advantages including shorter treatment timelines, streamlined manufacturing processes and potential cost reduction, positioning it as an important direction in cancer immunotherapy. Despite these advantages, *in vivo* CAR T-cell therapy also introduces new challenges, including delivery safety and engineering controllability, delivery system immunogenicity, limited persistence and solid tumor-specific barriers, all of which require further optimization to facilitate clinical translation. These major challenges are summarized in **Figure 3**.

### 4.1 Targeting, safety and controllability of delivery systems

Achieving specific delivery of CAR transgenes to T cells — particularly to the abundant resting T cells *in vivo* — represents a core technical challenge in the *in vivo* CAR T-cell field (61, 92). Many viral and non-viral delivery systems exhibit higher transduction efficiency for activated T cells, whereas resting T cells are generally more difficult to transduce or edit efficiently (112), further limiting the efficiency of *in vivo* CAR T-cell engineering.

Non-specific delivery may lead to CAR expression in non-target cells, potentially causing toxicity or immune escape (113). A representative case involved a patient with B-cell acute lymphoblastic leukemia (B-ALL) receiving anti-CD19 CAR T-cell therapy, in which a leukemia B cell was accidentally transduced during CAR T-cell manufacturing. This cell expressed the CAR and caused cis masking of the CD19 epitope, ultimately resulting in antigen escape and treatment resistance (114). Furthermore, integrating viral vectors carry a risk of insertional mutagenesis, which may cause cellular dysfunction or even oncogenicity (115, 116). To address these issues, various optimization strategies have been developed. Site-specific gene editing for precise integration is considered an important direction to simultaneously reduce risks of ectopic expression and random integration. For example, Nyberg et al. developed a two-vector *in vivo* gene editing system, using an enveloped delivery vehicle (EDV) displaying anti-CD3 antibodies to deliver CRISPR–Cas9 ribonucleoprotein, together with a human T cell-tropic AAV variant (AAV-hT7) obtained through directed evolution to deliver the CAR DNA donor template (117). AAV-hT7 not only exhibits enhanced transduction efficiency for human T cells but also partially evades pre-existing neutralizing antibodies in humans, thereby improving *in vivo* delivery efficiency. Notably, this CAR donor lacks an exogenous promoter; only cells that take up both vectors and achieve precise integration into the TRAC locus through homology-directed repair (HDR) can express the CAR. This design substantially enhances T-cell specificity of CAR expression and reduces risks of ectopic expression in non-T cells and random integration. Nevertheless, because HDR depends on cell cycle and DNA repair status, its *in vivo* editing efficiency remains limited, and low-frequency non-homologous integration events may still occur in theory (112, 118).

Beyond precision integration strategies, optimizing regulatory elements in viral vectors can further improve expression specificity. For instance, EsoBiotec has developed a T cell-specific promoter derived from human endogenous regulatory elements that enhances selective CAR expression in T cells and reduces ectopic expression in non-target cells. Additionally, unlike conventional ex vivo CAR T-cell manufacturing, *in vivo* CAR T-cell therapy lacks pre-infusion selection and quality release steps for engineered cells (119). Consequently, transduction efficiency, CAR copy number, phenotypic composition of cells, and *in vivo* expansion kinetics are difficult to control precisely (120). Achieving predictable regulation of CAR expression levels and numbers of engineered T cells remains a major challenge limiting clinical translation of *in vivo* CAR T-cell therapy.

Beyond preventive strategies, researchers have also attempted to introduce “suicide switches” to enhance safety. For example, drug-inducible suicide systems such as inducible caspase-9 (iCasp9) and herpes simplex virus type 1 thymidine kinase (HSV-TK) can trigger CAR T-cell apoptosis through exogenous drugs upon severe toxicity, providing a salvage clearance mechanism (121, 122).

### 4.2 Immunogenicity of delivery systems

Delivery systems themselves possess inherent immunogenicity, rendering them susceptible to recognition and clearance by pre-existing antibodies, the complement system and innate immune mechanisms *in vivo*, which reduces vector transduction efficiency and limits repeat administration (123, 124). For viral vectors, immunogenicity primarily arises from capsid or envelope proteins, as well as xenogenic domains contained in the transgene product — for example, scFv regions derived from murine antibodies (125, 126). These immune responses not only reduce transduction efficiency but may also affect treatment persistence and overall safety.

Moreover, the physicochemical properties of nanoparticles — such as size, surface charge and lipid composition — significantly influence complement activation and inflammatory responses, thereby indirectly affecting the risk of adverse events (127). To address these issues, multiple strategies to reduce immunogenicity have been proposed, including the development of low-immunogenicity or humanized viral capsids, engineered envelope proteins, optimized CAR architecture, increased nucleic acid chemical modification and optimized LNP formulations (128–131). These strategies offer new directions for improving *in vivo* CAR T-cell delivery efficiency and enabling repeat administration.

### 4.3 Persistence of *in vivo* CAR T cells

Although *in vivo* CAR T-cell therapy avoids conventional ex vivo expansion procedures, the challenge of insufficient CAR T-cell persistence remains. The long-term efficacy of CAR T cells is influenced by multiple factors, including CAR structural design, T-cell differentiation status, sustained antigen stimulation and T cell exhaustion (132). From a CAR structural perspective, the affinity of the extracellular antigen-binding domain influences signaling strength and duration, with excessively high affinity potentially leading to chronic activation and T-cell exhaustion (133). Co-stimulatory domains also have important effects on CAR T-cell persistence, with the CD28 intracellular domain tending to promote rapid effector function, whereas 4-1BB is generally associated with more durable survival and memory phenotypes (134). In addition, the hinge and transmembrane regions influence CAR stability, signaling intensity and *in vivo* function (135), although the underlying mechanisms remain incompletely understood.

From a delivery system perspective, the duration of CAR expression varies markedly among different vectors. Integrating LVs enable stable genomic integration and thus long-term CAR expression (59, 136). By contrast, non-integrating nanocarriers such as mRNA–LNPs typically support only transient expression. Although transient expression may limit CAR T-cell persistence, it allows more flexible dose regulation through repeat administration and may reduce toxicities associated with sustained chronic activation (137). Different delivery platforms thus present distinct trade-offs among persistence, safety, and controllability, and achieving the optimal balance requires further investigation.

### 4.4 Challenges of *in vivo* CAR T cells in solid tumors

Although many obstacles also exist in conventional ex vivo CAR T-cell therapy, the application of *in vivo* CAR T-cell therapy in solid tumors faces additional complex limitations (138). The physical barriers, immunosuppressive tumor microenvironment (TME) and antigen heterogeneity characteristic of solid tumors further impair the expansion, infiltration and sustained killing capacity of *in vivo*-generated CAR T cells.

#### 4.4.1 Physical barriers and impaired T-cell infiltration

CAR T cells must effectively reach the tumor site to exert their cytotoxic function. However, abnormal vascular structures and dense extracellular matrix (ECM) in solid tumors constitute important physical barriers. Aberrant tumor vasculature not only leads to tissue hypoxia and promotes recruitment of immunosuppressive cells but also downregulates adhesion molecules such as VCAM-1 and ICAM-1, thereby limiting T-cell extravasation (139–141). Furthermore, the fibrotic matrix dominated by cancer-associated fibroblasts (CAFs) further hinders CAR T-cell infiltration into the tumor core (142). In hepatocellular carcinoma, for example, extensive deposition of hyaluronan and collagen in the ECM creates a pronounced fibrotic barrier that compromises CAR T-cell infiltration (143, 144).

#### 4.4.2 Immunosuppressive tumor microenvironment

The solid tumor TME is a highly immunosuppressive ecosystem that impairs CAR T-cell function at multiple levels. Inhibitory cell populations such as tumor-associated macrophages (TAMs), myeloid-derived suppressor cells (MDSCs), regulatory T cells (Tregs) and CAFs directly suppress CAR T-cell activity through the secretion of inhibitory factors including TGF-β and IL-10 (145–147). These cellular and soluble immunosuppressive mechanisms collectively restrict CAR T-cell activation, expansion, persistence, and cytotoxic function within solid tumors. T cell exhaustion is considered a major factor limiting the efficacy of CAR T-cell therapy in solid tumors.

#### 4.4.3 Metabolic barriers and T-cell dysfunction

Beyond cellular immunosuppression, solid tumors impose spatially heterogeneous metabolic constraints on CAR T cells (148, 149). Rapidly proliferating tumor and stromal cells consume large amounts of glucose, glutamine, and essential or conditionally essential amino acids, including arginine, tryptophan, and cystine, thereby restricting the substrates required for CAR T-cell activation, proliferation, and biosynthesis (148–152). Enhanced tumor glycolysis further promotes lactate accumulation and extracellular acidification, which impair T-cell receptor signaling, cytokine production, and cytotoxic activity (150, 153, 154). Amino-acid catabolism can simultaneously generate suppressive metabolites; for example, tryptophan depletion and kynurenine accumulation through the IDO pathway inhibit T-cell proliferation and promote dysfunctional or exhausted states (148, 150). Extracellular adenosine generated through the CD39/CD73 axis provides an additional metabolic checkpoint by suppressing CAR T-cell effector function through A2A receptor signaling (155).

Hypoxia and oxidative stress further compromise CAR T-cell fitness. Reduced oxygen availability limits mitochondrial oxidative phosphorylation, whereas excessive reactive oxygen species disrupt redox homeostasis and decrease the production of IFN-γ, TNF-α, granzyme B, and perforin (149, 156–158). Persistent mitochondrial depolarization, impaired mitochondrial biogenesis, and reduced respiratory capacity restrict intratumoral migration, memory differentiation, self-renewal, and long-term persistence. These abnormalities may also promote cellular senescence, characterized by irreversible cell-cycle arrest and reduced proliferative and cytotoxic capacity. In parallel, dysregulated lipid metabolism—including altered cholesterol availability, oxidized-lipid accumulation, and lipid peroxidation—can impair T-cell membrane organization, survival, and effector function, while also influencing ferroptosis-related interactions between CAR T cells and tumor cells (149, 159–161).

Metabolic dysfunction is closely integrated with chronic antigen stimulation and inhibitory signaling. Sustained activation under hypoxic and nutrient-depleted conditions reinforces NFAT-, TOX-, and NR4A-associated exhaustion programs, accompanied by reduced effector function, impaired self-renewal, and increased expression of PD-1, TIM-3, and LAG-3 (148, 159). Moreover, hypoxia, lactate flux, acidity, and oxidative stress may fluctuate across circadian and tumor-regional contexts, creating time- and location-dependent differences in metabolic permissiveness (162). This issue may be particularly relevant to *in vivo* CAR T-cell therapy because CARs are installed directly into heterogeneous endogenous T-cell populations without prior ex vivo selection or metabolic conditioning. Consequently, variability in the baseline metabolic fitness of targeted T cells, together with platform-dependent CAR expression kinetics, may compound the metabolic barriers encountered after tumor infiltration (149, 150, 162).

#### 4.4.4 Antigen heterogeneity and tumor escape

Solid tumors typically exhibit substantial antigen heterogeneity, with marked variability in antigen expression density across different regions and clones (142). Unlike hematological malignancies with relatively uniform target expression, solid tumors comprise diverse subpopulations with distinct molecular and phenotypic characteristics. This heterogeneity can arise from multiple biological processes, including genetic instability, epigenetic alterations, and microenvironmental stresses, which collectively contribute to divergent antigen expression patterns among tumor cells (148). Moreover, antigen heterogeneity exists not only among different tumor lesions but also within individual tumors, resulting in spatial variations and dynamic changes in antigen availability (148). Under the selective pressure imposed by CAR T-cell therapy, tumor cells with low or absent target expression may preferentially survive and progressively expand, leading to antigen escape and disease relapse (148, 163). In addition to antigen downregulation or loss, other escape mechanisms, including antigen mutation, alternative splicing, epitope masking, and defects in antigen processing, may further compromise CAR T-cell recognition and cytotoxic activity (148). These challenges are also relevant to *in vivo*-generated CAR T cells, as antigen heterogeneity may limit tumor recognition and promote antigen escape.

#### 4.4.5 Potential enhancement strategies in solid tumors

In response to the above challenges, multiple enhancement strategies are being developed to improve the efficacy of *in vivo* CAR T-cell therapy. To improve tumor infiltration, local administration strategies can concentrate CAR T cells or delivery vehicles directly at the tumor site — for example, through intratumoral injection, intrapleural infusion or intraperitoneal administration — to partially circumvent physical barriers and chemokine deficits in solid tumors (164, 165). Hepatocellular carcinoma (HCC) is considered a promising application scenario for *in vivo* CAR T-cell therapy, owing to its relatively well-defined local delivery routes and extensive research foundation in liver-targeted delivery (166). Moreover, liver-enriched delivery systems such as modified LNPs may further improve local delivery efficiency and reduce systemic toxicity risks (167). However, current related research remains largely at an early exploratory stage, and the safety, delivery specificity, and long-term efficacy of these approaches require further validation.

To reshape the immunosuppressive and metabolically hostile tumor microenvironment, multiple complementary approaches are being explored, including suppression of MDSC and Treg activity, blockade of the PD-1/PD-L1 axis, cytokine-based regulation, and metabolic intervention (168, 169). Unlike conventionally manufactured CAR T-cell products, *in vivo* CAR T-cell platforms bypass the ex vivo manufacturing stage during which metabolic conditioning and enrichment of metabolically favorable T-cell subsets can be performed (150). Metabolic optimization of *in vivo*-generated CAR T cells may therefore be achieved through construct-level engineering, co-delivery of regulatory cargos, or combination therapies administered *in vivo*. In conventionally manufactured CAR T cells, representative metabolic engineering strategies have included enhancing nutrient acquisition through glucose transporters such as GLUT1 or GLUT3 and amino-acid transporters such as LAT1 or xCT, restoring arginine biosynthesis through argininosuccinate synthase 1 or ornithine transcarbamylase, promoting mitochondrial biogenesis through regulators such as PGC-1α, and degrading immunosuppressive metabolites through enzymes such as kynureninase or D-2-hydroxyglutarate dehydrogenase (150, 170–172). Collectively, these strategies provide a mechanistic basis and a repertoire of candidate modules for developing metabolically optimized *in vivo* CAR T-cell platforms.

Complementary extrinsic interventions may remodel the metabolic microenvironment by targeting tumor-derived lactate, the IDO1–kynurenine pathway, the CD39/CD73–adenosine axis, or dysregulated lipid metabolism. For example, in glioblastoma models, LDHA inhibition with oxamate reduced tumor-derived lactate and lactylation-associated expression of CD39, CD73, and CCR8, thereby enhancing CAR T-cell infiltration, immune activation, and tumor control (173). The distinct kinetic properties of *in vivo* delivery platforms create additional opportunities for integrating metabolic regulation. The transient and repeat-dose characteristics of mRNA–LNP systems may enable temporally adjustable delivery of metabolic-regulatory or anti-exhaustion cargos, whereas T-cell-targeted integrating LVs could support more sustained expression of selected metabolic-support modules. In parallel, circadian-aware vector administration or pulsatile CAR expression has been proposed as an emerging strategy to align CAR installation and effector activity with periods of greater metabolic fitness, vascular permissiveness, and immune competence (162). Together, these approaches highlight the potential of *in vivo* CAR T-cell platforms to integrate metabolic and temporal control and may offer greater flexibility than is readily achievable with a single infusion of conventionally manufactured CAR T cells.

In response to antigen heterogeneity and antigen escape, dual-targeting and multi-targeting CAR designs can broaden tumor antigen coverage and reduce the risk of antigen-negative escape (174, 175). In contrast, AND- and NOT-gated CAR designs are primarily intended to enhance tumor specificity and reduce the risk of off-tumor recognition through combinatorial antigen recognition and inhibitory signaling (174, 176). In addition, introducing artificial antigens onto the surface of tumor cells through viral, nanoparticle or bacterial delivery systems offers new approaches to address antigen loss and escape (177, 178). Furthermore, accumulating evidence indicates that the gut microbiota can influence CAR T-cell efficacy and toxicity by modulating host immune status (179, 180). Modulating gut microbiota composition may help enhance CAR T-cell expansion and antitumor effects while reducing the risk of adverse events such as CRS (181). Thus, individualized intervention strategies based on gut microbiota may represent an important direction for optimizing *in vivo* CAR T-cell therapy in the future.

## 5 Clinical research progress

With accumulating preclinical evidence and progressive maturation of delivery technologies, *in vivo* CAR T-cell therapy has begun to transition from the laboratory to human clinical trials. In recent years, multiple first-in-human studies have been initiated to preliminarily evaluate the safety, tolerability, and preliminary efficacy of lentiviral vector-based and LNP–mRNA formulations in patients with malignancies and autoimmune diseases. Below, we summarize the major clinical research progress in this field, and representative existing *in vivo* CAR T-cell products are listed in **Table 1**.

**Table 1:** Overview of existing *in vivo* CAR T-cell products.

Clinical trial ID	CAR T-cell product	Sponsor	Delivery vehicle	Target antigen	Conditions	Phase	Study status	Efficacy outcomes	Safety outcomes
NCT06528301 (185)	UB-VV111 ± Rapamycin	Umoja Biopharma	LV (CD3 scFv, CD80 and CD58)	CD19	LBCL/CLL	I	Recruiting	Not yet reported	Not yet reported
NCT06743503 (186)	UB-VV400 ± Rapamycin	Nanjing IASO	LV (CD3 scFv, CD80 and CD58)	CD22	Large B-cell Lymphoma	I	Recruiting	Not yet reported	Not yet reported
NCT07109986 (187)	UB-VV410	Nanjing IASO	LV (CD3 scFv, CD80 and CD58)	CD22	SLE/Lupus Nephritis	I	Recruiting	Not yet reported	Not yet reported
NCT06539338 (188)	INT2104	Interius BioTherapeutics	LV (CD7 scFv)	CD20	B-cell Cancers	I	Active, not recruiting	Not yet reported	Not yet reported
NCT07075185 (189)	KLN-1010	Kelonia Therapeutics	LV	BCMA	Multiple Myeloma	I	Recruiting	Interim analysis (n=6): all patients achieved objective responses and MRD-negative bone marrow status at month 1; complete resolution of extramedullary disease was observed in 1 patient.	Infusion-related reactions occurred in 3/6 patients; grade 2 CRS occurred in 4/6 patients; no ICANS or delayed neurotoxicity was observed.
NCT06791681 (182)	ESO-T01	Chunrui Li	LV	BCMA	Relapsed/Refractory Multiple Myeloma	I	Recruiting	Interim analysis (n=5): 4/5 patients achieved objective responses, including 3 stringent complete responses (sCRs); all evaluable responders were MRD-negative by day 60.	CRS occurred in 4/5 patients, including 3 grade 3 events; transient cytopenias, reversible liver-enzyme elevations, 3 grade 2 infections, and 1 grade 1 ICANS event were reported.
NCT06691685 (22)	ESO-T01	Huazhong University of Science and Technology	LV	BCMA	Multiple Myeloma	I	Recruiting	All four treated patients achieved objective responses, including 2 stringent complete responses (sCRs) and 2 partial responses (PRs).	CRS occurred in all 4 patients, including 3 grade 3 events; no dose-limiting toxicity (DLT) was reported.
NCT05969041 (190)	MT-302	Myeloid Therapeutics	LNP	TROP2	Epithelial Tumors	I	Recruiting	Among 27 treated patients, 1 confirmed PR was observed in a hormone receptor-positive breast cancer patient; CAR-positive myeloid-cell tumor infiltration and pharmacodynamic immune activation were demonstrated.	Low-grade CRS occurred in 51.9% of patients, with no grade ≥3 CRS; 1 grade 4 ICANS event occurred at 0.15 mg/kg; MTD without steroid premedication was 0.10 mg/kg.
NCT06478693 (191)	MT-303	Myeloid Therapeutics	LNP	GPC3	Liver cancer	I	Recruiting	Not yet reported	Not yet reported
NCT06890065 (192)	JY-231	Shenzhen Genocury	LV	CD19	B Acute Lymphoblastic Leukemia, B-Non Hodgkin Lymphoma	I	Recruiting	Not yet reported	Not yet reported
NCT06678282 (183, 184)	JY231	Shenzhen Genocury	LV	CD19	B-NHL, B-ALL	I	Recruiting	Case-level evidence: 1 patient with relapsed/refractory DLBCL achieved CR; cohort-level efficacy data not yet available.	Transient myelotoxicity and grade 1 CRS were reported; no ICANS or infection was observed.
NCT07002112 (193)	LIVTO-TaVec100	The First Affiliated Hospital with Nanjing Medical University	LV	CD19/​CD20	Relapsed/Refractory B-cell Malignancies	I	Recruiting	Interim analysis (n=12): ORR was 50.0% (6/12) and CR rate was 41.7% (5/12) across all dose levels; at dose level 2, ORR was 100% (6/6) and CR rate was 83.3% (5/6).	Infusion-related reactions occurred in 9/12 patients and CRS in 8/12 patients; all events were grade 1–2. No DLTs, serious adverse events, ICANS, non-ICANS neurotoxicity, or deaths were reported.
NCT06618313 (194)	JCKH-213	Beijing GoBroad Hospital	LNP- mRNA	CD19	B-NHL	I	Recruiting	Not yet reported	Not yet reported
NCT06917742 (195)	CPTX2309	Capstan Therapeutics	LNP- mRNA	CD19	Healthy Volunteers、Rheumatoid ArthritisSystemic、Lupus Erythematosus	I	Recruiting	Not yet reported	Not yet reported
NCT06801119 (23)	HN2301	Shenzhen MagicRNA Biotechnology Co., Ltd	T-cell-targeted LNP delivering CD19 CAR mRNA	CD19	SLE, SSc and RA	I	Interventional	Refractory-SLE cohort (n=5): 4-mg dose achieved complete peripheral B-cell depletion (<1 cell/μL); all patients showed reduced SLEDAI-2K scores at 3 months.	No grade ≥3 CRS or ICANS was observed; off-target CAR expression was below 10%.

CRS, cytokine release syndrome; ICANS, immune effector cell-associated neurotoxicity syndrome; MRD, minimal residual disease; sCR, stringent complete response; CR, complete response; PR, partial response; ORR, objective response rate; DLT, dose-limiting toxicity; MTD, maximum tolerated dose; SLEDAI-2K, Systemic Lupus Erythematosus Disease Activity Index 2000; DLBCL, diffuse large B-cell lymphoma; SAE, serious adverse event.

Umoja Biopharma has advanced several programs based on its VivoVec platform (described above) into clinical development. The company currently has three Phase I trials underway: UB-VV111 targeting CD19 (NCT06528301) and UB-VV400 targeting CD22 (NCT06743503) for the treatment of relapsed/refractory B-cell malignancies, and UB-VV410 targeting CD22 (NCT07109986) for active refractory systemic lupus erythematosus (SLE) or lupus nephritis.

Another clinical study evaluated the lentiviral vector-mediated *in vivo* CAR T-cell therapy ESO-T01 in relapsed/refractory multiple myeloma. Early published data from four patients showed two achieving stringent complete remission (sCR) and two achieving partial remission. Subsequently updated clinical data further demonstrated that among five treated patients, four achieved objective responses, including three with sCR. From a safety perspective, no dose-limiting toxicities were observed, but cytokine release syndrome occurred in four patients, including three grade 3 events; transient cytopenias and other adverse events were also reported. Concurrently, the investigators detected CAR T cells in peripheral blood and tumor-associated tissues, supporting that ESO-T01 enables *in vivo* CAR T-cell generation and expansion (22, 182). In addition, early publicly available clinical information for JY231 has also shown preliminary activity. Disclosed case and conference data indicate that one patient with multiply relapsed B-cell acute lymphoblastic leukemia achieved minimal residual disease-negative complete remission within one month of treatment, and another patient with diffuse large B-cell lymphoma and high tumor burden also attained complete remission. As these data are derived primarily from early clinical disclosures, their efficacy and safety profiles require further confirmation through additional formally published studies (183, 184).

In the solid tumor arena, publicly available human clinical data for *in vivo* CAR T-cell therapy remain lacking. Notably, *in vivo* CAR engineering strategies have begun to expand to other immune cell types. For example, MT-302 and MT-303, which target TROP2 and GPC3 respectively, are based on mRNA–LNP platforms and primarily function through *in vivo* reprogramming of myeloid cells; early clinical studies have been initiated in patients with solid tumors. Although these strategies fall outside the narrow definition of *in vivo* CAR T-cell therapy, their clinical advancement suggests that the translational potential of *in vivo* CAR immune-cell engineering in solid tumors is progressively emerging (196). Concurrently, *in vivo* CAR T-cell technology is being actively extended to the treatment of autoimmune diseases. An ongoing Phase I clinical trial (NCT06917742) is evaluating the safety and tolerability of CPTX2309, an mRNA–LNP-based *in vivo* CAR T-cell product, in healthy volunteers as well as in patients with moderate-to-severe rheumatoid arthritis (RA) or SLE. Another study provides the first human feasibility evidence for *in vivo* CAR T-cell therapy in SLE. Five patients with refractory SLE received HN2301, an engineered LNP targeting CD8^+^ T cells that encapsulates mRNA encoding an anti-CD19 CAR. CD8^+^ CD19 CAR T cells were detectable in peripheral blood as early as 6 hours after infusion, with both CD19 CAR T-cell and CAR mRNA levels peaking at this time point and returning to baseline within 2–3 days; off-target expression was below 10%. No grade 3 or 4 cytokine release syndrome or immune effector cell-associated neurotoxicity syndrome was observed. Within 6 hours of treatment, patients receiving the 2 mg dose showed a marked reduction in circulating B cells, whereas those receiving the 4 mg dose achieved complete B cell depletion (<1 B cell/μL). At the 3-month follow-up, all five patients showed a reduction in SLEDAI-2K scores, indicating decreased disease activity (23). Collectively, *in vivo* CAR T-cell therapy has demonstrated preliminary feasibility, acceptable early-stage safety, and encouraging efficacy signals in hematologic malignancies and autoimmune diseases. Although current studies have limited sample sizes and short follow-up durations, the data obtained thus far lay a foundation for further development of this technology. Larger-scale clinical trials are needed to confirm long-term efficacy, optimal dosing regimens, and applicability across different indications.

## 6 Conclusion and perspectives

CAR T-cell therapy has demonstrated remarkable clinical value in hematologic malignancies and has profoundly reshaped the landscape of cancer immunotherapy. However, the complex manufacturing processes, lengthy production timelines, and high costs inherent to conventional ex vivo manufacturing approaches still limit its broader accessibility and application. Against this backdrop, *in vivo* CAR T-cell therapy—which enables the *in situ* engineering of endogenous T cells through the direct delivery of CAR-encoding information within the body—offers a potentially transformative paradigm for overcoming these bottlenecks. Leveraging viral vectors, lipid nanoparticles, and other engineered delivery platforms, *in vivo* CAR T-cell therapy holds the promise of circumventing the cumbersome steps of ex vivo cell isolation, transduction, and expansion, thereby shortening treatment timelines, reducing production costs, and advancing CAR T-cell therapy toward a more standardized, rapidly deployable, and accessible modality.

Despite its promise, *in vivo* CAR T-cell therapy remains at an early stage of development, and its clinical translation depends on resolving key issues related to delivery specificity, engineering controllability, long-term safety, and therapeutic persistence. In complex *in vivo* settings, especially in solid tumors, achieving a balance among efficient generation, precise regulation, and durable efficacy will remain a major direction for future technological optimization. Looking ahead, continued refinement of targeted delivery systems, gene-editing tools, regulatable CAR designs, and multifunctional delivery platforms is expected to progressively improve the specificity, efficacy, and safety of *in vivo* CAR T-cell therapy. At the same time, in-depth investigations into pharmacokinetics, tissue distribution, long-term toxicity, and immune safety, together with the development of scalable manufacturing processes, regulatory frameworks, and ethical guidelines, will be essential for advancing this technology toward clinical application. Overall, *in vivo* CAR T-cell therapy represents an important extension of the conventional adoptive cell therapy paradigm, holds the potential to reshape the way CAR T-cell therapies are generated and applied, and may become a major direction in the future development of cell-based therapeutics.
